# Surface and Electrical Characterization of Ag/AgCl Pseudo-Reference Electrodes Manufactured with Commercially Available PCB Technologies

**DOI:** 10.3390/s150818102

**Published:** 2015-07-24

**Authors:** Despina Moschou, Tatiana Trantidou, Anna Regoutz, Daniela Carta, Hywel Morgan, Themistoklis Prodromakis

**Affiliations:** Nanoelectronics and Nanotechnology Research Group, Southampton Nanofabrication Centre, Electronics and Computer Science, University of Southampton, SO17 1BJ Southampton, UK; E-Mails: T.Trantidou@soton.ac.uk (T.T.); a.regoutz@imperial.ac.uk (A.R.); D.Carta@soton.ac.uk (D.C.); hm@ecs.soton.ac.uk (H.M.); T.Prodromakis@soton.ac.uk (T.P.)

**Keywords:** integrated reference electrode, PCB technology, Ag/AgCl, biosensing, Lab-on-Chip, Lab-on-PCB

## Abstract

Lab-on-Chip is a technology that could potentially revolutionize medical Point-of-Care diagnostics. Considerable research effort is focused towards innovating production technologies that will make commercial upscaling financially viable. Printed circuit board manufacturing techniques offer several prospects in this field. Here, we present a novel approach to manufacturing Printed Circuit Board (PCB)-based Ag/AgCl reference electrodes, an essential component of biosensors. Our prototypes were characterized both structurally and electrically. Scanning Electron Microscopy (SEM) and X-Ray Photoelectron Spectroscopy (XPS) were employed to evaluate the electrode surface characteristics. Electrical characterization was performed to determine stability and pH dependency. Finally, we demonstrate utilization along with PCB pH sensors, as a step towards a fully integrated PCB platform, comparing performance with discrete commercial reference electrodes.

## 1. Introduction

Lab-on-a-Chip (LoC) technology has been established as one of the most promising candidates for revolutionizing medicine, owing to its inherent Point-Of-Care (PoC) capabilities: advanced functionality, low sample volumes, rapid results, and increased portability [1,2]. While for the past years research has focused on improving LoC performance, the current bottleneck in its commercial adoption is the development of cost-effective upscaling strategy [3,4]. Semiconductor manufacturing techniques have been heavily employed for diagnostic platforms [5,6], however, there is no standardized reliable procedure to integrate microfluidics in an economically viable fashion. On the other hand, microfluidics can be manufactured with alternative processes and materials, such as glass [1], polymer [7] and even paper substrates [8], but the integration of electronics is currently challenging. Printed Circuit Board (PCB) manufacturing, although primarily aimed at consumer electronics applications, has recently been adopted as an alternative promising approach, facilitating the effortless integration of electronics and microfluidics rendering a new era: Lab-on-PCB platforms [9,10,11].

Several LoC components and prototypes have been demonstrated on Printed Circuit Boards (PCBs) [12,13,14], including chemical sensors. In order to acquire sensitive and reliable sensor readings, stable integrated reference electrodes are required [15,16,17,18,19,20]. In this direction, Cranny *et al*. [21] have shown screen-printed Ag/AgCl pseudo-reference electrodes for soil salt measurements, while Bhavsar *et al*. [22] utilized PCB fabricated Ag/AgCl pseudo-reference electrodes combined with electrochemical biosensors for cytokine detection. So far, however, studies have focused on the end application rather than on an investigation of the physical and electrical characteristics of the reference electrodes.

Whilst there are several techniques to deposit Ag on substrates in the research lab (e.g., E-gun evaporation, sputtering), in PCB industries such techniques are not available. In the present work, Ag/AgCl pseudo-reference electrodes have been fabricated solely via commercially available techniques used routinely in PCB manufacturing for applying a Ag finish to standard electronic PCBs. The geometry of our electrodes has been optimized to serve as a component of more complex Lab-on-PCB systems. In this paper, we have investigated their physical characteristics, electrical stability and pH dependence, whilst benchmarking performance with commercially available reference electrodes in a pH sensing experiment.

## 2. Experimental Section

Our prototype pseudo-reference electrodes comprise an array of 80 vias of different diameters, ranging from 300 to 1000 μm. This configuration was chosen to match a previously fabricated pH sensing electrode array platform [23,24]. Utilizing a via geometry is expected to enable the exploitation of the reference electrodes as sample outlets, when subsequently integrated within a microfluidic network [25]. All prototyped reference electrodes are equipotential and electrical connectivity is established through standard PCB headers soldered onto the boards.

A 2 × 4 cm^2^ prototype reference electrode platform was micromachined with commercially available PCB technologies from Newbury Electronics Ltd, UK. After patterning the copper layers (35 μm thick) and forming the via holes, solder paste was applied, prior to immersion Ag coating of the patterned Cu electrodes. The Ag coating was performed with the MacDermid Sterling^TM^ Silver [26,27] standardized industrial process for PCB electroless immersion silver plating. Vertical industrial polymer tanks were used, incorporating both mechanical agitation of the chemical solutions and a constant vibration of the boards to ensure small via conformal plating. The cleaned PCBs were first immersed in 100 L of the Sterling 2.0 Predip solution (93.4% Water, 5% Sterling Silver Part B proprietary mixture, 1.6% Concentrated Nitric Acid) at 38 °C for 30 s. Following the Predip, the PCBs were immediately immersed in 130 L of the Sterling 2.0 Silver solution (85.5% Water, 10% Sterling Silver Part B proprietary mixture, 2% Concentrated Nitric Acid, 2.5% Sterling Silver Part A proprietary mixture) at 52 °C for 120 s. All concentrations are volume per volume.

In order for the deposition to be successful both solutions need to be maintained within MacDermid’s specifications (Table 1) in terms of (a) Acid Normality = $\frac{Volume_{NaOH}\times \left(N_{NaOH}\right)}{Solution\;volume}$; (b) Chelator Molarity = (mL of Copper Nitrate) × (M of Copper Nitrate) × 0.05; (c) Copper concentration; (d) Silver concentration; and (e) pH. Acid Normality and pH is maintained within the specifications by adding Nitric Acid, Chelator Molarity by adding Sterling^TM^ Silver Part B and Silver concentration by adding Sterling^TM^ Silver Part A. If the copper concentration exceeds the limit, the solutions are replaced with fresh ones.

**Table 1:** MacDermid solution specifications.

Solution	Acid Normality	Chelator Molarity	Copper mg/L	Silver g/L	pH	Temperature °C
Sterling^TM^ Predip	0.2–0.3 N	0.01–0.02 M	<1000	N/A	<1.8	38
Sterling^TM^ Silver	0.4–0.6 N	0.02–0.04 M	<3000	0.6–0.9	<1.8	52

This process results in conformally Ag-plated copper patterns, thus avoiding the contact of copper structures with biological samples. Copper is a well-known antimicrobial material, and could unduly influence the assays.

The Ag coated PCBs were subsequently rinsed with water, dried, cleaned with acetone/IPA and immersed for 10 min in a sodium hypochlorite NaOCl solution (NaOCl, Sigma-Aldrich, reagent grade, available chlorine 4.00%–4.99%), utilizing the silver layer to produce the AgCl layer, thus formulating the Ag/AgCl pseudo-reference electrodes. 

A dual-beam focused ion beam/scanning electron microscope system (Zeiss NVision 40 FIB/FEGSEM) equipped with a gas injection system (GIS) was used to record SEM images and for cutting of FIB cross-sections. SEM images were recorded at an accelerating voltage of 5 kV. Prior to performing FIB cross-sections, an electron beam-induced tungsten protective layer was deposited on the top of the electrodes in order to minimize damage caused by the gallium ions in the subsequent ion beam-induced tungsten deposition step.

The surface of the Ag-coated Cu contacts before and after chlorination was characterized using XPS. The spectra were recorded on a Thermo Scientific Theta Probe Angle-Resolved X-ray Photoelectron Spectrometer (ARXPS) system (base pressure 2 × 10^−9^ mbar), incorporating a monochromated Al Kα X-ray source (hν = 1486.6 eV) and a 180° double focusing hemispherical analyzer with two-dimensional PARXPS detector. The X-ray source was operated at 6.7 mA emission current and 15 kV anode bias. Data was collected using a 200 × 200 µm^2^ X-ray spot and a pass energy of 200 eV. A flood gun was used to minimize sample charging and spectra were aligned assuming the C 1s core line to be at a binding energy of 285.0 eV.

The PCB pseudo-reference electrodes were electrically characterized by measuring their potential (V_pcb_) difference versus a commercial (V_commercial_) Ag/AgCl discrete reference electrode (CH Instruments Inc., CHI111, Austin, TX, USA) directly after fabrication, when dipped in three different pH buffer solutions (Hanna Instruments, HI-7004, 7007, 7010): pH = 4, 7 and 10. The potential difference values were recorded every minute, through a Picoscope 2205 data logger (Pico Technology) with PicoLog software over one day.

**Figure 1:**
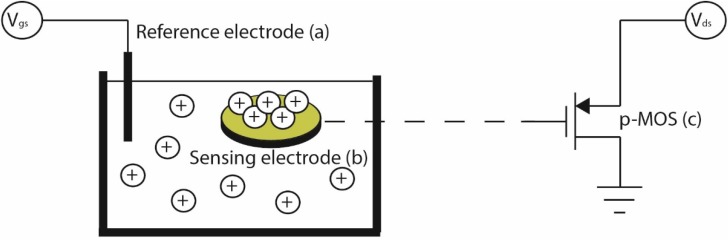
Schematic of the pH sensing apparatus.

The performance of the PCB pseudo-reference electrodes for chemical sensing was benchmarked against commercial reference electrodes and silver wires [28] that were chlorinated with a similar procedure and are often exploited in custom biosensing platforms [23]. In this experiment, we employed an extended gate PCB-based biosensing platform, as reported in [24], where the ion selective membrane was a 200 nm thick indium tin oxide (ITO) film (90:10 = In_2_O_3_:SnO_2_) that was sputtered on top of Au platted Cu electrodes. According to the ionic strength of the liquid, H^+^ binds to the ITO membrane surface, deposited on top of the Au plated PCB sensing sites. These charged sites are electrically coupled to the metal–oxide–semiconductor field-effect transistor (MOSFET) floating gates (Figure 1), causing a shift in their turn-on voltage V_on_. The electrical characterization of the chemical sensors was performed with a Keithley semiconductor characterization system (SCS-4200). An array of p-type MOSFETs (Figure 1, point (c)) was mounted on a custom design instrumentation system [23]. The extended gate PCB sensors (Figure 1, point (b)) were remotely connected to the gates of the discrete transistors through a ribbon cable. The transistor drain was biased continuously at 0.5 V and the source was connected to ground (0 V), while the gate bias was applied to the respective (commercial Ag/AgCl, Ag/AgCl wire, PCB Ag/AgCl) reference electrodes (Figure 1, point (a)). The gate voltage was swept from −3 V to 0 V and the respective drain current-gate voltage (I_ds_-V_gs_) transfer characteristics were recorded. All experiments were performed at room temperature inside a Faraday cage to minimize the influence of external noise sources.

## 3. Results and Discussion

### 3.1. Surface Characteristics

**Figure 2:**
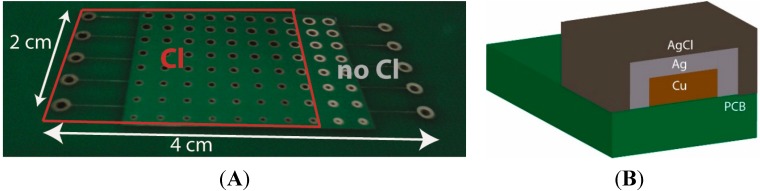
(**A**) Image of the PCB (Printed Circuit Board) pseudo-reference electrodes before (area on the right) and after (squared area on the left) NaOCl treatment; and (**B**) schematic cross-section of the Ag/AgCl reference electrode stack.

**Figure 3:**
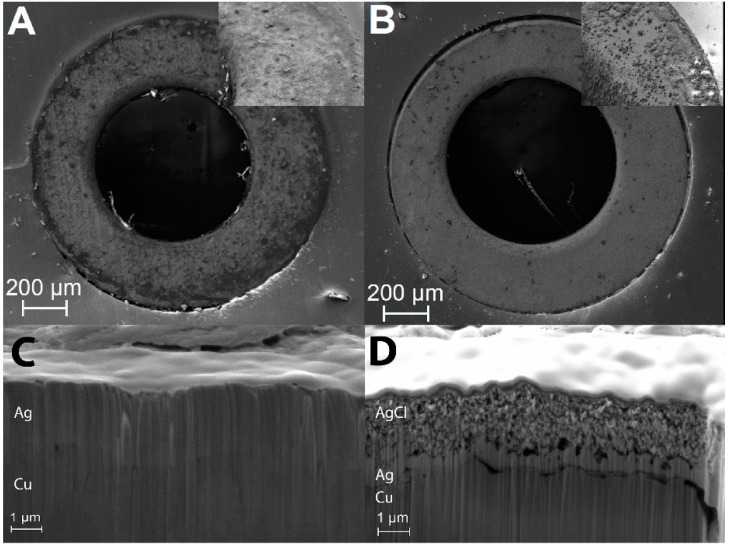
SEM images of the Ag-coated contacts (**A**) before and (**B**) after chlorination and FIB cross-sections of the Ag-coated contacts (**C**) before and (**D**) after chlorination.

The fabricated PCBs were characterized before and after NaOCl treatment to verify chlorination of the Ag layer (Figure 2A). The color change of the electrode vias from bright silver (Ag) to brown (AgCl) indicates that an AgCl layer was formed on top of the Ag plating (Figure 2B), consistent with previous studies [28]. Qualitative indication of the formation of the AgCl deposition layer is also given by comparing the SEM images of the non-chlorinated and chlorinated electrodes, as shown in Figure 3A,B, respectively. An additional layer is visible in the case of the chlorinated electrode, and it is even more evident in the magnified image shown in the inset of Figure 3B. The formation of the AgCl layer in the chlorinated sample was also confirmed via FIB cross-section imaging. The FIB cut of the non-chlorinated sample is shown in Figure 3C. The thickness of the Ag layer was confirmed to be in the range of 1.4 μm and can clearly be observed on top of the Cu contact. The FIB cut of the chlorinated sample, shown in Figure 3D, shows two layers having different morphology. The thickness of the Ag layer, deposited on the Cu contact is not homogeneous and has a maximum thickness of 650 nm after chlorination. The AgCl layer was identified to be in the range of 1.5 μm.

**Figure 4:**
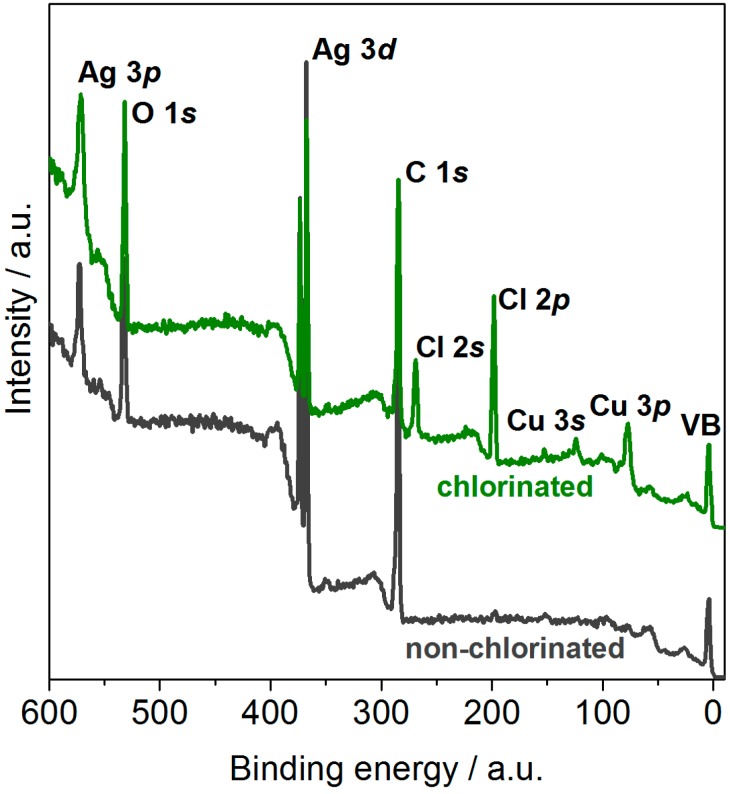
XPS survey spectra of the Ag-coated contacts before and after chlorination. All core lines are indicated.

In order to evaluate the surface properties of both non-chlorinated and chlorinated Ag-coated Cu contacts, XPS spectra of non-chlorinated and chlorinated Ag-coated Cu contacts were collected (see Figure 4). The untreated contacts show Ag 3d and 3p as well as O 1s and C 1s core lines. As XPS is a surface sensitive method with penetration depths of a few nm, the Cu beneath the Ag coating is not observed in the untreated sample. However, Cu 2p, 3p and 3s core lines are present after chlorination. The Cu layer is not completely covered by the Ag and upon exposure to sodium hypochlorite it undergoes the following transition: Cu + NaOCl → CuO + NaCl. During the preparation, it is possible that CuO is re-deposited on the surface of the Ag electrode surface. This is supported by the energy of the Cu 2p line being at the characteristic energy for CuO and satellite structures at higher binding energies of the main core lines being consistent with CuO [29,30]. The successful chlorination of the contacts is confirmed by the presence of Cl 2p and Cl 2s core lines.

### 3.2. Characterization of Electrode Stability

**Figure 5:**
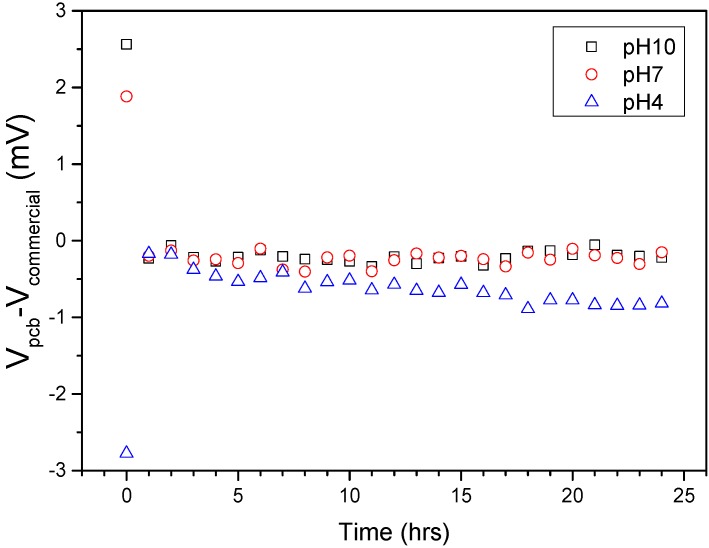
Voltage difference between PCB (Printed Circuit Board) pseudo-reference electrodes and commercial Ag/AgCl reference electrodes V_pcb_-V_commercial_ evolution with time at different pH values.

After confirming, the formation of a Ag/AgCl structure, we verified electrode stability by comparing the open-circuit potential with commercial Ag/AgCl reference electrodes. The voltage difference (V_pcb_-V_commercial_) evolution in time can be seen in Figure 5. As previously reported, upon initial immersion of the reference electrodes in any solution, a set-up time is required in order for the open circuit potential to stabilize [19]. For all three buffer solutions, our PCB electrodes also require an initial set-up time to stabilize (Figure 5). The electrodes demonstrate a very stable electrical behavior in the long term (drift < 1 mV/24 h). Furthermore, they only differ by approximately 1 mV from commercial Ag/AgCl electrodes, irrespective of the buffer solution pH. For the acidic buffer (pH = 4) a more pronounced drift is observed, attributed to larger AgCl layer dissociation [16,18].

**Figure 6:**
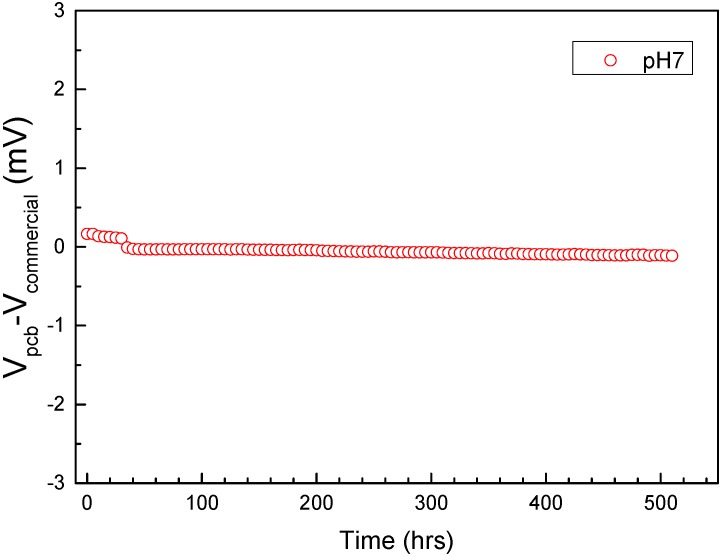
Voltage difference between PCB (Printed Circuit Board) pseudo-reference electrodes and commercial Ag/AgCl reference electrodes V_pcb_-V_commercial_ evolution over 500 h (20 day) at neutral pH.

Since close to neutral buffers are most commonly used in biological analysis, the long term stability of the PCB reference electrodes was recorded over an even longer period of time at pH = 7. Figure 6 clearly demonstrates that even for a total period of 500 h (20 day) the electrodes remain stable (<1 mV/20 day), with the most important drift taking place the first 48 h of continuous operation.

**Figure 7:**
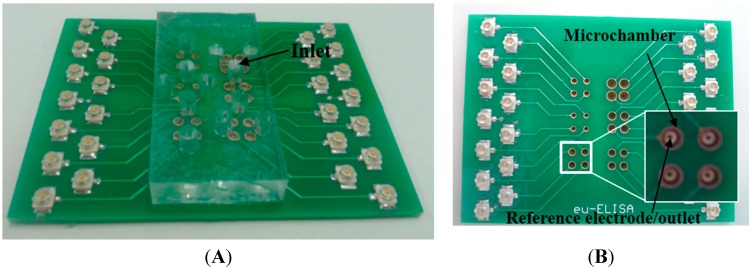
(**A**) Lab-on-PCB (Printed Circuit Board) integrating PMMA (Poly-methyl methacrylate) microfluidics and PCB microchambers and reference electrodes. (**B**) Two layer PCB before the attachment of microfluidics, comprising PCB reference electrodes in the bottom layer and microchambers in the top layer.

**Figure 8:**
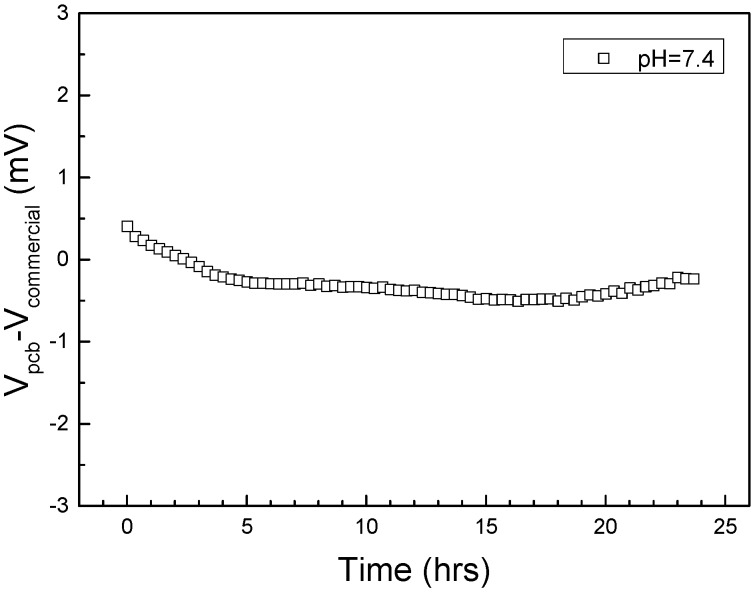
Voltage difference between PCB (Printed Circuit Board) pseudo-reference electrodes and commercial Ag/AgCl reference electrodes V_pcb_-V_commercial_ evolution with time under constant flow (2.5 μL/min) of HEPES buffer (pH = 7.4).

The PCB reference electrodes described in this work are intended to be used as components in integrated Lab-on-PCB systems, hence will need to be as electrically stable when utilizing biological buffers flowing through them. Therefore, a microfluidic delivery network was laser micromachined (Epilog Laser) in PMMA (Figure 7A) and attached with double sided tape on a double layer PCB (Figure 7B); the first layer of the PCB comprises the reference electrodes and the second layer has cylindrical, gold-plated microchambers (V_chamber_ = 1 μL). HEPES buffer (pH = 7.4) was injected via the inlet placed on the PMMA, with the reference electrode via serving as the microchamber outlet. The buffer was continuously flowed through the reference electrode for 24 h using a laboratory syringe pump (Chemyx Inc., Fusion 200, Stafford, TX, USA) at a flow rate of 2.5 μL/min. The open circuit voltage of the PCB reference electrodes against a commercial Ag/AgCl reference electrode was recorded for these 24 h (Figure 8). It can be observed that, even under constant flow, the PCB reference electrodes demonstrate excellent stability (<1 mV/day), however this drift is slightly higher compared to the static immersion tests, probably due to faster AgCl dissolution under constant flow.

### 3.3. Electrode Performance for pH Sensing

After extracting the physical and electrical stability characteristics of the electrodes, their performance in chemical sensing was evaluated. In particular, ITO PCB pH sensitive passive electrodes were utilized. The sensor PCB was immersed in three different pH buffer solutions and the respective p-MOS transfer curves were recorded while sweeping the gate voltage applied to the reference electrode. Four different reference electrodes were studied for comparison: a commercial Ag/AgCl, a NaOCl treated Ag wire and Ag/AgCl PCB reference electrodes.

**Figure 9:**
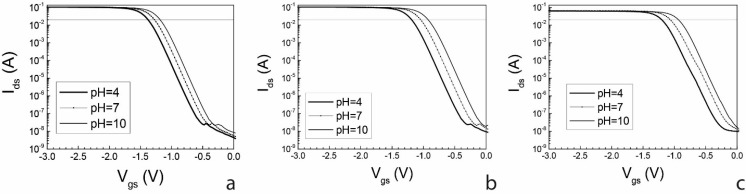
Extended gate transfer I_ds_-V_gs_ characteristics of ITO PCB (Indium Tin Oxide Printed Circuit Board) sensors for (**a**) commercial Ag/AgCl; (**b**) chlorinated Ag wire; and (**c**) Ag/AgCl PCB reference electrodes.

As Figure 9 shows, a positive shift of the transfer curves with increasing pH for all reference electrodes was observed. The translation of the curves is parallel, so that the turn-on voltage V_on_ of each extended gate transistor can be used to indicate the solution pH. In the present case, V_on_ is defined as the gate voltage for which the drain current I_ds_ is equal to 20 mA.

**Figure 10:**
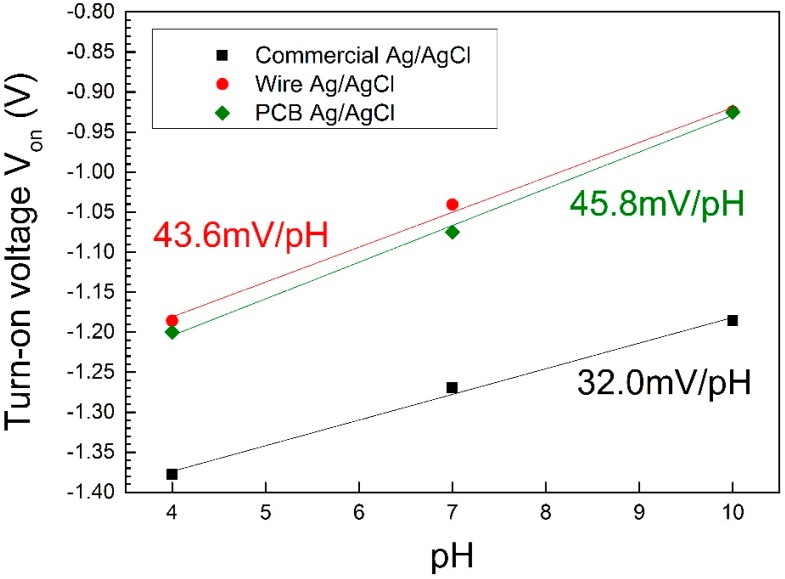
ITO PCB (Indium Tin Oxide Printed Circuit Board) sensor pH sensitivity (turn-on voltage V_on_ shift) for different types of reference electrodes (commercial, wire, PCB).

Figure 10 shows V_on_ for all three studied reference electrodes against pH values. For the commercial reference electrodes we observe a linear relationship between V_on_ and pH, with a sensitivity of 32 mV/pH. For both pseudo-reference electrodes, the linear relationship is again verified, featuring a sensitivity of 45.8 mV/pH and 43.6 mV/pH in the case of PCB and Ag wire, respectively. As previously reported (Figure 5), for more acidic samples we observe lower open circuit potential values for PCBs (V_pcb_) than for commercial ones (V_commercial_), thus causing the small difference in sensitivity.

## 4. Conclusions

In this work, we have demonstrated that stable Ag/AgCl pseudo-reference electrodes can be fabricated solely utilizing techniques available by PCB manufacturers. This opens the way for PCB compatible versions of components for biosensing platforms, complementing the development of PCB biosensors and Lab-on-PCB systems. Successful chlorination of the electrodes was proven by surface characterization techniques (SEM and XPS). The AgCl layer was estimated to be in the range 1.5 μm thick by FIB cross-sections imaging. PCB reference electrodes demonstrated excellent long-term stability (<1 mV/24 h). PCB reference electrodes were combined with PCB pH sensors and successfully tested, giving a sensitivity of 45.8 mV/pH, which is comparable to the commercial Ag/AgCl electrodes. We aim to integrate these stable PCB integrated reference electrodes that can serve both as reference electrodes and as fluidic outlets for PCB microfluidics in a Lab-on-PCB platform currently under development to provide a fully integrated immunodiagnostic chip.
